# Sonography as a Diagnostic Tool in Midurethral Sling Complications: A Narrative Review

**DOI:** 10.3390/jcm13082336

**Published:** 2024-04-18

**Authors:** Aneta Zwierzchowska, Paweł Tomasik, Edyta Horosz, Ewa Barcz

**Affiliations:** Department of Gynaecology and Obstetrics, Medical Faculty Collegium Medicum, University of Cardinal Stefan Wyszynski, 01-938 Warsaw, Poland; a.zwierzchowska@uksw.edu.pl (A.Z.); p.tomasik@uksw.edu.pl (P.T.); e.horosz@uksw.edu.pl (E.H.)

**Keywords:** midurethral sling, stress urinary incontinence, sonography

## Abstract

Despite the established safety and efficacy of midurethral slings (MUS), which are the current gold standard treatment for stress urinary incontinence (SUI), the potential for postoperative complications remains a significant concern for both healthcare professionals and patients. Meanwhile, sonography has emerged as a significant diagnostic tool in urogynecology, and one of the applications of this imaging modality may be the evaluation of complications arising from MUS procedures. This review, based on a comprehensive literature search, focuses on the use of pelvic floor ultrasound (US) in the context of MUS complications. It includes analyses of randomized controlled trials, prospective, and retrospective studies, covering preoperative and postoperative investigations, to assess complications such as persistent and recurrent SUI, urinary retention and obstructive voiding, de novo urgency/overactive bladder, vaginal exposure, sling erosion, pain, and hematoma. The review critically examines the existing literature, with a particular focus on recent publications. Despite the variability in findings, it appears that for each of the discussed complications, the application of pelvic floor US can significantly support the diagnostic and therapeutic process. The paper also identifies potential future directions for the development of US applications in diagnosing MUS complications.

## 1. Introduction

The tension-free vaginal tape (TVT) introduced by Ulmsten and Petros in 1995 emerged as a pivotal advancement in treating stress urinary incontinence (SUI), shifting from the previously considered gold-standard Burch colposuspension. TVT proved to be a simple, safe, and minimally invasive procedure, characterized by high effectiveness, as evidenced by an 81–93% cure rate over long-term follow-up [1,2,3]. In 2001, Delorme introduced a novel approach for tape insertion via the obturator foramen [4]. Comparative studies demonstrated that both the transobturator tape (TOT) and TVT offer comparable efficacy to Burch colposuspension, with cure rates of 84–95% for TOT and 80–94% for TVT at 1-year follow-up, yet with the added benefits of shorter operation times and hospital stays [5,6,7,8].

Over two decades of research have affirmed the safety and efficacy of MUS procedures, with widespread endorsement from professional and governmental bodies worldwide [9]. Nonetheless, the possibility of postoperative complications presents a substantial challenge for healthcare professionals and patients alike [10]. The most common adverse effects include persistent SUI, urinary retention, de novo urgency, pain, vaginal exposure of the sling, and hematoma.

A recently published study in Denmark involving 329 women over five years found that while 85% reported no complications post-MUS, 15% experienced one or more complications [11]. This emphasizes the importance of careful patient selection, detailed preoperative counseling, meticulous surgical execution, and vigilant postoperative care to minimize complications and optimize patient outcomes [12].

Meanwhile, in the field of urogynecology, sonographic imaging has become an indispensable diagnostic tool, both in the preoperative diagnostic process and for detecting complications associated with various procedures [13].

Within the scope of urogynecological diagnostics, various sonographic techniques are utilized:

- transvaginal and transrectal approaches;
- external methods: perineal, introital, and transabdominal approaches [14].

These methods provide consistent, reproducible results, allowing for the visualization of key anatomical structures such as the bladder, urethra, and pelvic floor muscles.

The US examination technique in urogynecology has been standardized and detailed in the regularly updated recommendations of AIUM/IUGA as well as German gynecological and sonographic societies.

**Figure 1:**
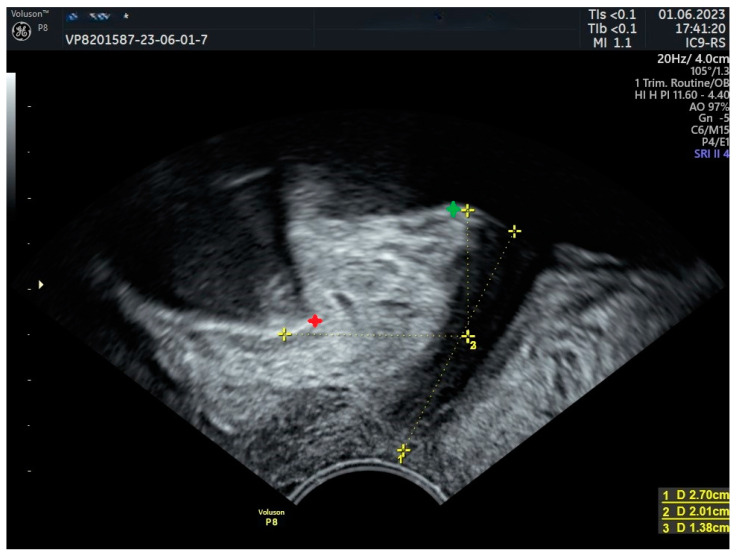
Urethral length and bladder neck position at rest. Green marker—bladder neck; red marker—symphysis pubis; yellow mark 1: urethral length, yellow mark 2: bladder neck position over the lower edge of symphysis pubis.

Urinary incontinence and the presence of synthetic implants, including MUS, are among the indications for performing US examinations of the pelvic floor [15]. For introital US examination, a vaginal probe at 2–10 MHz is utilized, while for perineal US, a sector scanner with a frequency of 5–9 MHz is preferred. In both techniques, the patient is placed in a lithotomy position and the probe is positioned at the introitus, ensuring high-resolution imaging with minimal contact pressure and aligning the transducer perpendicular to the body axis. Optimal visualization, especially in diagnostics related to incontinence, necessitates a bladder filling of approximately 300 mL, as this volume significantly influences the imaging of the bladder trigone and neck position. For the sake of consistency in studies and publications, images are standardized with cranial structures displayed at the top, caudal structures at the bottom, ventral structures on the right, and dorsal structures on the left side of the image, although deviations from this format may occur in clinical practice. During the examination, several key parameters are assessed: the volume of residual urine to evaluate bladder emptying, the urethra’s length and mobility, and the bladder neck’s position at rest (Figure 1) and on exertion/coughing. The presence of bladder neck funneling is also evaluated, alongside the inspection of periurethral tissue for any pathologies like diverticula or cysts. The coordination and movement of the levator ani muscle complex are observed, as well as the thickness of the detrusor wall. Postoperative imaging, focusing on the correct placement and condition of surgical interventions such as tapes or bulking agents and identifying complications such as hematomas, has also become a standard [14].

To identify a sling implant using the US, it is necessary to visualize the implant as a brief hyperechoic line or curve, approximately 1 cm in length, in the mid-sagittal plane. In the orthogonal planes, the implant should appear as a longer (>2.5 cm) curvilinear hyperechoic structure [16].

Pelvic floor sonography appears to be a relatively simple examination. A recent study demonstrated that trainees are able to rapidly acquire the skills necessary to recognize anatomical landmarks and evaluate MUS using sonography with a proficiency comparable to that of an experienced attending radiologist [17].

US techniques have also proven to be highly valuable in the context of MUS procedures. Increasing evidence suggests that the tape’s placement in relation to the urethra plays a crucial role in determining the success of the sling [18,19]. Research indicates that for optimal outcomes, the tape should ideally be positioned at 50 to 75% of the urethral length, starting from the bladder neck to the external urethral meatus [19,20,21]. This specific area aligns with the urethra’s high pressure zone, located between the point of maximum urethral closure pressure and the urethral knee [22].

Sonography has also been utilized in both pre- and postoperative evaluations of SUI.

When a sonographic examination of the pelvic floor is performed, the lower part of the symphysis usually serves as a reference point. As mentioned above, typically, patients are positioned in the lithotomy position [23]. However, bladder neck descent is more pronounced when standing, and the examination may be repeated if urethral hypermobility is not observed in the lithotomy position. Bladder filling volume also influences bladder neck descent during US [24]. Consistent bladder filling volume is essential for accurate measurements in the same patient to evaluate bladder neck descent or urethral hypermobility [25].

**Figure 2:**
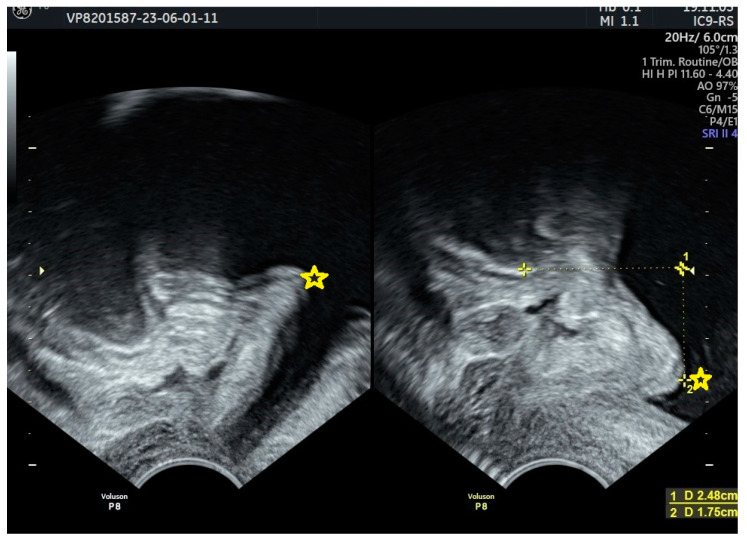
Bladder neck descent and funneling during the Valsalva maneuver. Yellow star—bladder neck; yellow mark 1: position of the bladder neck below lower edge of symphysis pubis.

It has been demonstrated that women with SUI exhibit a greater preoperative descent of the urethrovesical junction during the Valsalva maneuver compared to those without SUI. Funneling of the vesical neck is another characteristic associated with SUI (Figure 2).

These findings are recognized and may be employed in assessing patients for whom MUS is considered, as they corroborate the clinical diagnosis of genuine SUI [26].

**Figure 3:**
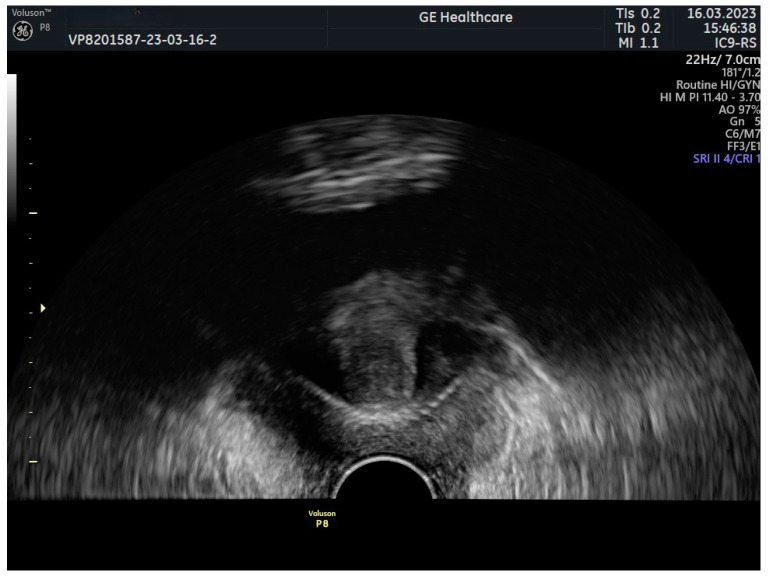
TVT—coronal view.

The two most prevalent sling types, TOT and TVT, are easily distinguishable in US by their configurations. In coronal view, TVT exhibits a “U” shape, while TOT presents as a broader “hammock”, with arms extending horizontally through the obturator foramina [25] (Figure 3).

Beyond structural delineation, sonography is increasingly recognized for its effectiveness in assessing complications from MUS procedures. Its ability to offer dynamic, non-invasive visualization of synthetic slings in various settings enhances its value and is especially useful in cases lacking comprehensive surgical history [27].

The current paper endeavors to explore the increasing significance of sonography in the identification and management of MUS complications. Through a detailed examination of the potentials of sonographic imaging in this context, we aim to highlight its importance and possible impact on enhancing patient care in the realm of synthetic sling-related complications.

## 2. Materials and Methods

A literature search using PubMed was performed in January 2024, focusing on randomized controlled trials (RCTs) as well as prospective and retrospective studies related to US application in MUS, including preoperative and postoperative assessments, as well as sling complications. The search terms utilized were “ultrasound”, “mid-urethral sling”, “TOT”, “TVT”, and “complications of mid-urethral slings”, either individually or in combination. Selection was limited to articles in English and those considered pertinent to the review. The literature of the last 20 years was analyzed, resulting in 141 articles. After further eliminating case reports and case series articles, 81 were included in the analysis.

## 3. MUS Complications and the Diagnostic Role of PF Sonography

### 3.1. Persistent and Recurrent SUI

Persistent SUI, or treatment failure, is considered by some authors a complication of the MUS procedure.

In a multicenter study, with patient-reported outcomes serving as the basis for evaluation, the TOT method achieved a subjective effectiveness rate of 84%, whereas patients who underwent the TVT procedure had an 80% cure rate. Follow-up visits were conducted more than six months post-operation [28]. Other analyses yielded similar reports [29,30]. In a comparative meta-analysis of the TVT and TOT techniques, the TVT group demonstrated slightly higher objective (86% vs. 84%) and subjective (78% vs. 74%) continence rates, but the aggregate continence rates were comparatively similar [31]. In an older randomized equivalence, the 12-month objective success rates for the TVT and TOT procedures equaled 80.8% and 77.7% [32]. Anger et al. found that within the initial year following MUS, 8% of patients encountered treatment failure [33]. In a prospective observational study aimed at evaluating the cure rates and late complications associated with the TVT, it was found that 81.3% of women were completely cured, as assessed seven years post-surgery [34]. In the study performed by Kociszewski et al., who evaluated solely patients with complications of MUS, persistent SUI or insufficient treatment effect was the second most common complication of MUS [35].

While managing SUI, some authors claim that the precise placement of the sling is paramount for its effectiveness and that sonography plays a crucial role in evaluating patients experiencing SUI after the MUS procedure [36]. Indeed, US imaging provides a non-invasive means to examine various critical parameters that will be further discussed.

**Figure 4:**
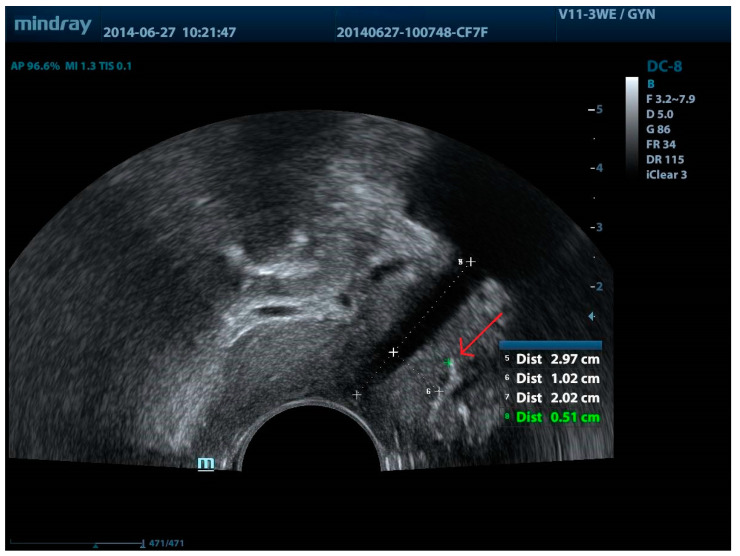
MUS located too far from the urethra (greater sling-LSM distance). Red arrow—sling. 5: urethral length, 6: distance from the closer margin of the sling to LSM, 7: sling distance from the bladder neck; 8: the distance between the closest margin of the sling and LSM.

The aforementioned study illustrated that persistent SUI correlates with the sling’s position under the urethra and in relation to the bladder neck. Among women with persistent SUI and sling exposure, the distances between the sling and the longitudinal smooth muscle (LSM) (the distance between the tape and the urethra) were found to be 3.6 mm and 4.6 mm, respectively. Hence, greater sling-LSM distance was associated with an increased risk of persistent SUI (Figure 4). The authors also showed that persistent SUI was linked to the proximity of the sling to the bladder neck, with observations indicating that in the affected patients, the sling was situated closer to this structure [35].

Similarly, in a detailed analysis conducted by Jiang et al., who used transrectal sonography in women who had undergone MUS implantation, it was found that individuals with the sling positioned at the bladder neck experienced a significantly higher recurrence rate of SUI compared to women whose sling was placed suburethrally at various lengths along the urethra [37]. On the other hand, in a recent prospective study involving 92 patients, US using an endovaginal probe was employed in 92 women post-MUS and the study found no correlation between sling positioning in relation to the bladder neck (proximal vs. middle vs. distal) and sling failure, which was defined by both subjective and objective indications of persistent or worsened SUI, recurrent SUI, or retreatment for SUI. It must be noted, however, that the study group was heterogenic, with 84.8% having undergone the TOT procedure and 15.2% the TVT [38]. In a more nuanced investigation by Bogusiewicz et al., sonography was conducted in a cohort of patients exhibiting persistent SUI post-MUS. The study found the median tape placement to be at 35.8% along the urethral length. In 73.8% of the women, the tapes were situated beneath the proximal half of the urethra, whereas only in 21.3% of the patients, the tapes were located between 50% and 75% of the urethral length, aligning with the urethra’s high-pressure zone. The authors concluded that placing the tape beyond this zone might be identified as a reason for the failure of a suburethral sling [39]. In a prospective study, Flock et al. investigated over 300 patients to examine the placement of the TVT. Utilizing introital US, they also found that a sling positioned either too close to the bladder neck or too far towards the urethral meatus, marked by positions outside the 40 to 81% range of the urethral length while at rest, was significantly linked to persistent SUI. In contrast to what was reported by the authors of the previously cited analysis, however, no association between the position of the sling in relation to the urethral lumen and the persistence of SUI was demonstrated [40]. Tunitsky-Bitton et al. conducted transperineal and introital pelvic floor US in patients who had previously undergone an MUS procedure. Regardless of the type of sling, there was a significant relationship between the position of the sling being closer to the urethrovesical junction and the severity of incontinence. Slings positioned closer to the external urethral meatus were associated with greater incontinence severity. Of note, a different measurement performed by the authors, i.e., the distance between the sling and the symphysis pubis at rest or during maximal Valsalva maneuver, did not show a significant correlation with scores on either of the urinary symptom scales [41].

**Figure 5:**
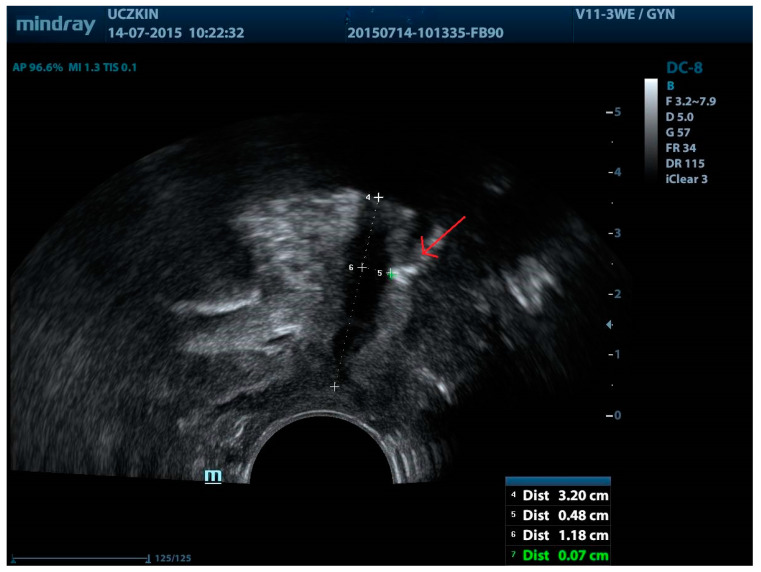
TVT positioned too close to the bladder neck. Red arrow—sling. 4 urethral length; 6 sling distance from the bladder neck; 7 sling distance from LSM.

Figure 5 demonstrates a sling placed too close to the bladder neck.

Another study utilized US to examine the placement of TOT in relation to the length of the urethra. The determination of treatment success was based on urine leakage during the cough stress test at 250 mL and responses to the UDI-6 questionnaire. It was noted that the sling’s placement in the midurethral region during maximal Valsalva maneuver occurred in 84% of women who remained continent compared to only 12% of those who experienced treatment failure [42]. Older research comparing the outcomes of the TVT versus TOT approach did not reveal a statistically significant association between the position of both sling types and the rates of treatment success. Still, a trend akin to that observed in the abovementioned studies was discernible, with the average proximity of the tapes to the bladder neck being closer in patients who experienced treatment failure. The lack of a significant difference might be attributable to the limited sample size in this particular study [43]. Finally, in a recent meta-analysis designed to systematically review the impact of suburethral tape location (both TVT and TOT) on surgical outcomes, it was found that the position of TVT at the urethral midpoint significantly correlated with higher cure rates compared to placement at the distal urethra. Of note, the cure rates for the retropubic tapes located in the proximal urethra were comparable to those observed with distal urethral placement. In relation to the TOT method, positioning the tape at the urethral midpoint significantly improved cure rates compared to proximal placement. Patients with the tape positioned at the urethral midpoint had similar cure rates to those with tape at the distal urethra and location of the tape in the distal urethra resulted in higher cure rates than proximal placement. This analysis included data from seven prospective cohort studies and two retrospective studies [44].

These findings delineate the critical impact of sling placement on the therapeutic outcomes of SUI interventions as well as the role of sonography in the diagnosis of persistent SUI. On the other hand, Dietz et al. did not demonstrate any correlation between the sling position and its effectiveness [45]. Similar results were obtained by Manonai et al., who focused on clinical symptoms and US data in women who underwent 3D endovaginal US. In the latter research, among patients who had their sling positioned under the proximal urethra (less than the 40th percentile), 56.2% reported moderate to severe disturbance from urine leakage related to physical activity. 37.5% of patients with the sling located under the midurethra (40th to 70th percentile) or the distal urethra (beyond the 70th percentile) experienced similarly bothersome symptoms. The authors found no significant difference in the level of discomfort reported between these groups [46].

Another publication was released that reconciles seemingly contradictory observations from the aforementioned studies. In this study, dynamic translabial US was employed on 110 women who had received the “out-in” TOT procedure for SUI. The sling’s position was categorized as “proximal” if situated within the initial 0–40% of the urethral length from the bladder neck, “mid-urethral” if within the 40–60% region, and “distal” if along the latter 60–100% segment. Concordance of urethral movement with the sling was defined as the sling’s location remaining constant at maximum Valsalva compared to its position at rest. Conversely, discordance was noted when these positions varied. The researchers observed that, six months post-surgery, women with persistent incontinence had their slings positioned more commonly in either a more proximal (31.3% vs. 1.6%) or distal (68.8% vs. 3.1%) location compared to those who were continent. Additionally, in these incontinent patients, the sling exhibited discordant movement with the urethra, alongside asymmetry of the arm and bladder neck funneling. Conversely, continent patients showed a significant reduction in urethrocele grade both at rest and during Valsalva maneuver [47]. The aforementioned study focusing on dynamic evaluation of sling functionality also indicated that the synchronization between urethral movement and the sling’s position was paramount in achieving continence. All patients displaying this congruent pattern of tape behavior were continent one year post-surgery, compared to 34% of women with a suboptimal outcome [42].

The existing research also addresses another important US finding in the context of diagnosing and treating SUI: funneling of the bladder neck, also known as urethral funneling. Funneling is defined as opening of the urethral internal orifice, assessed usually during the Valsalva maneuver [48].

In an analysis of patients visiting a urogynecological clinic, all of whom underwent pelvic floor US, it was discovered that in every case of genuine SUI confirmed by a positive cough test, the length of urethral funneling during the Valsalva maneuver exceeded 50% of the total urethral length, indicating extensive urethral funneling. Conversely, in 83.7% of the women who did not exhibit SUI, urethral funneling was not observed [49]. In a retrospective analysis, it was shown that TVT placement significantly reduces bladder neck funneling, whereas correction of funneling correlated with increased success rates. Patients without postoperative funneling had a cure rate of 96.2% compared to 57.5% in patients with continued postoperative funneling. Similarly, patients without preoperative funneling had a cure rate of 96.6%, versus 77.5% for those with preoperative funneling. However, no relationship was found between the distance of TVT from the urethra or its position relative to the urethra and funneling (the authors mentioned that the TVT was positioned below the upper third of the urethra in only two cases, making it difficult to conclude any relationship with funneling). The resolution of funneling post-surgery is considered a positive sonographic indicator, implying stabilization of the endopelvic fascia and paraurethral support structures [26,50].

Other parameters related to the position and behavior of the suburethral tape, observable in US examinations, have also been deemed significant by some authors in the context of the tape’s efficacy.

In the previously cited study conducted by Kociszewski et al., it was found that the cure rate was 100% among women classified as Group I, who had a tape that was “stretched out” at rest and assumed a C-shape during straining. In contrast, among patients with tape shapes falling into Groups II and III, where the tape either remained parallel to the urethral lumen or was already “C-shaped” at rest, 39% experienced incontinence.

The authors referenced earlier also utilized US to observe the sling’s behavior during the Valsalva maneuver, employing a methodology akin to the one described above. They noted that the ability of the sling to deform on the Valsalva maneuver was visible in 70% of women who remained continent, compared to just 10% of individuals who experienced persistent or recurrent SUI one year post-operation [42].

Chene and colleagues conducted a study involving US to compare various parameters among patients who had undergone TVT, TOT, and TVT-O. They utilized a transvaginal probe to precisely determine the position of the tape and the bladder neck relative to the infero-posterior margin of the symphysis pubis in a strictly sagittal plane. Additionally, they performed a dynamic assessment of the tape’s behavior in a strict frontal plane, considering the angulation between the two tape arms, both at rest and during activities such as the Valsalva maneuver and maximum pelvic floor muscle contraction. The resting position of the tape (within the TOT and TVT-O groups exclusively) demonstrated a correlation with clinical improvement. It was observed that a greater angle of the tape at rest was notably linked to the recurrence of SUI [51].

Importantly, US can reveal other contributory factors to persistent SUI. For instance, an open bladder neck or proximal urethra observed on imaging might indicate the presence of intrinsic urethral sphincter deficiency, a condition beyond the mere mechanics of sling placement. Additionally, the lack of dynamic compression observed in imaging might suggest either a loosening of the sling, possibly due to an early return to vigorous activities post-operation, or a technical failure during the initial placement [13].

Recent findings indicate that the sling’s placement tends to remain constant throughout observation [25], mirroring the observations by Majkusiak et al., who also reported stability in the sling’s location over time [27]. Interestingly, other studies validated this observation for the TOT approach, although the TOT method involves a more extensive sub-urethral dissection compared to TVT or TVT-O, and therefore one might raise concerns regarding the potential for tape migration towards either the bladder neck or the urethral meatus (this situation would most likely occur directly after the procedure, as a result of the tape shifting when the patient is upright) [51].

When analyzing the potential causes of improper (proximal) tape placement, various factors must be considered. According to the technique described by Ulmsten for the suburethral sling placement, the vaginal incision should begin approximately 1 cm from the external urethral meatus [1]. Improper tape location may be linked to incorrect preparation of the tape tunnel. However, it is important to reference the study by Pomian et al., which highlighted significant variations in urethral length across the population, ranging from 19 to 45 mm with a normal distribution of this parameter. Obese patients exhibited significantly longer urethras compared to non-obese individuals, and the number of vaginal deliveries was associated with shorter urethral lengths [52].

Although the outcomes of various studies are not unequivocal, there are discernible trends that underscore the significance of pelvic floor US as an indispensable diagnostic resource. It undoubtedly provides detailed insights into the multifaceted dynamics influencing the persistence of SUI following sling placement. Still, it must be stressed that not every case of persistent or recurrent SUI can be directly attributed to a factor visible on US. In research analyzing the failure risk factors of MUS procedures (both TVT and TOT), multivariate analysis identified BMI > 25 (OR, 2.9), mixed incontinence (OR, 2.4), prior continence surgery (OR, 2.2), intrinsic sphincter deficiency (OR, 1.9), and diabetes mellitus (OR, 1.8) as significant independent predictors of MUS failure [53]. Evaluating success based solely on the patient’s subjective assessment presents challenges; from our experience in interviewing women with urinary incontinence, it is often difficult for them to distinguish whether SUI or OAB predominates. Yet, as demonstrated, OAB is an independent risk factor for the failure of anti-incontinence surgery. This is supported by further analysis: Holmgren et al. found that the cure rate for TVT in patients with mixed incontinence was lower than in those with SUI alone. Women with SUI maintained a cure rate of 85% from two to eight years post-procedure. In contrast, women with mixed incontinence had a persistent cure rate of 60% up to four years postoperatively, which then decreased to 30% from four to eight years after surgery [54]. Despite the lack of consensus on the optimal management strategy for patients with persistent/recurrent SUI after MUS placement, other authors agree that conducting a pelvic floor US before deciding on the next management strategy is advisable [55].

### 3.2. Urinary Retention and Obstructive Voiding

Urinary retention and difficulties in voiding are recognized as potential complications arising from surgical interventions for SUI. Any surgical intervention targeting SUI inherently introduces an element of obstruction, as these procedures are designed to improve urethral closure. The MUS functions more as a compensatory mechanism than a reconstructive one, leading to continence that is attained with a certain degree of obstruction [56]. Through its corrective action on the deficiency in urethral support, the sling establishes a new distal urethral suspension mechanism for achieving continence. This mechanism creates dynamic urethral resistance, which becomes active during stress-inducing situations but does not disrupt normal urethral function during rest periods [57].

A comprehensive overview revealed varying prevalence rates of urinary retention post-MUS procedures. The incidence for TVT slings ranged from 2.5% to 19.5%, whereas for TOT slings, it was noted to be between 1.5% and 8.6% [58]. Klutke et al. in their study of 600 TVT patients found a 2.8% rate of retention or obstructive symptoms [59]. Anger et al., analyzing sling surgeries in over 65-year-old females, observed a 6.9% incidence of outlet obstruction among 1356 procedures within a year [33]. A Danish study evaluating 329 TVT surgeries over five years noted urinary retention as the most common complication, affecting 7% of the women [11]. A broader review highlighted that urinary retention related to the tape occurs in about 14.4% of patients, particularly with the retropubic approach [12]. Other authors reported retention rates varying between 2.2% and 16% [59,60,61], and in a TVT-focused study, 23% experienced retention, with 83% resolving within three months [62]. Abouassaly et al. reported 19.5% retention in 241 TVT patients, with 68% resolving in less than 48 h [63]. In a study comparing TVT and SPARC (a top-down TVT MUS), no cases of retention were found in the TVT group, versus 6.5% in the SPARC group [64]. In a randomized clinical trial of 92 patients undergoing TVT or TOT, 37.5% in the TVT group and 9.7% in the TOT group experienced retention up to seven days post-surgery, with no significant difference in cases lasting over seven days [65]. Chene et al.’s analysis of TVT, TOT, and TVT-O patients showed de novo voiding difficulties in 20%, 13%, and 13%, respectively [51].

Kociszewski et al.’s examination of MUS complications revealed that 40% of patients experienced urinary retention, with 16% also reporting overflow incontinence. A crucial finding was the median tape-LSM distance of 1.1 mm in patients with urinary retention [35]. Further, in another study, Kociszewski et al. associated a tape-urethra distance of less than 2 mm with obstructive complications, observing a 2.8-fold increased risk of such issues in these cases, with a median distance of 1.3 mm during a 48-month follow-up [66]. Reich et al. noted urinary retention over 200 mL in two cases from both TVT and TOT groups, with introital US showing a tape-urethra distance under 2 mm [67]. Accordingly, Viereck et al. also reported a correlation between close tape-LSM distance and voiding dysfunction. The authors conducted a retrospective study using medical records from women who underwent sling incision procedures. Before the incision, 61% of the women exhibited impaired bladder emptying, with an average residual urine volume of 210 mL. A significant difference was observed in the distance between the sling and the LSM, with a cutoff of less than 3 mm distinguishing patients experiencing voiding dysfunction from those without. Specifically, the median distance for those affected was 1.5 mm, compared to 3.6 mm for those without dysfunction [68].

Larson et al. explored Lower Urinary Tract Symptoms (LUTS) in women post-MUS placement, using both transabdominal and transvaginal sonography for evaluation. They categorized patients into three groups (I, II, and III) based on tape characteristics and behavior—as previously described by Kociszewski et al. [66]. Urodynamic studies indicated that Group III exhibited significantly higher maximum detrusor pressure compared to Groups I and II, hinting at obstructive voiding patterns. Furthermore, Group III’s sling positioning was significantly closer to the proximal region than in the other groups, although post-void residual volume data was not reported [69].

**Figure 6:**
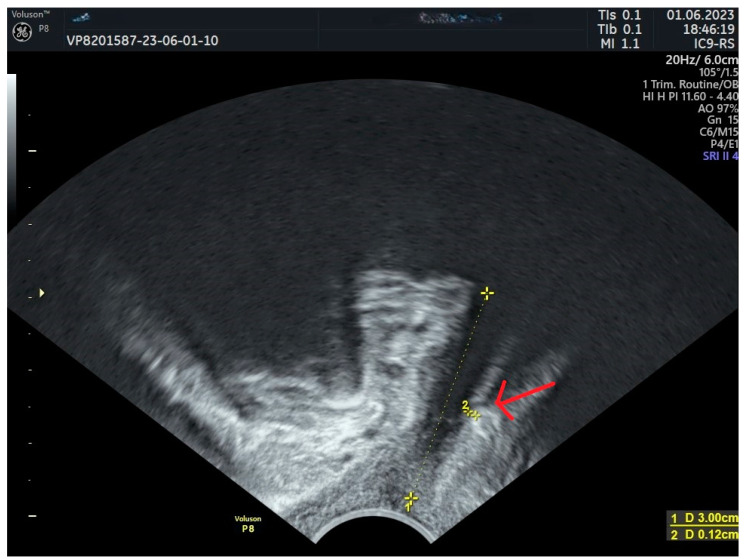
Close tape-LSM distance, C-shaped tape. Red arrow—tape. 1: urethral length; 2: sling distance from LSM.

Figure 6 demonstrates a sling that is placed too close to the urethra and at the same time exhibits Group II behavior (C-shaped both at rest and during the Valsalva maneuver).

Similarly, Takacs et al. investigated LUTS in women post-MUS placement using dynamic 2D transperineal pelvic US, by grouping patients based on the same sling dynamics during Valsalva maneuvers. Urodynamic tests showed increased detrusor pressure in Group III compared to Groups I and II, with a significant association persisting even after adjusting for factors like urethral length and sling-to-urethra distance. Despite no significant correlation between sling dynamics and various postvoid residual thresholds, dynamic 2D transperineal US was deemed effective in identifying women at risk of high-pressure voiding and diagnosing bladder outlet obstruction [70].

Finally, a recent publication also evaluated the same patterns of sling-shape behavior in the context of complications post-MUS. It was found that the likelihood of experiencing voiding dysfunction was significantly higher in women categorized as having a Group III MUS shape (C-shaped both at rest and during Valsalva), with 64% of these patients affected. Conversely, voiding dysfunction was observed in only 36% of the patients who were part of Group I, where the MUS remained flat during both rest and Valsalva maneuver [71].

In a previously cited analysis, the angulations associated with clinical and ultrasonographic successes were compared with those corresponding to postoperative voiding disorders. A closer angulation during the Valsalva maneuver was found to be linked with postoperative voiding disorders. Due to a substantial difference in angulation at rest observed in the TVT group, the analysis was limited to the TOT and TVT-O groups [51].

As seen above, researches reveal that closer sling placement to the urethra and certain sling dynamics are indicative of higher risks for obstructive complications. These insights emphasize the utility of a detailed sonographic evaluation in identifying patients at risk of adverse outcomes, thereby guiding clinical decisions to optimize patient care post-sling implantation.

### 3.3. De Novo Urgency/OAB

As mentioned earlier, Stanford et al. reviewed complications of suburethral sling procedures across 20 studies involving 1950 patients, finding a 15.4% overall incidence of de novo Overactive Bladder (OAB) symptoms, with rates ranging from 1.7% to 42% due to diverse outcome measurement methods [12]. Other analyses have similarly highlighted postoperative urgency as a common complication, with incidence rates from 5.9% to 25% for retropubic techniques and 0% to 15.6% for TOT [58]. A systematic review and meta-analysis reported de novo urgency occurrence between 3.1% and 29% [72]. In the aforementioned Danish study, ten of the enrolled women, accounting for 3%, developed de novo urgency [11]. Anger et al. noted a 15.2% incidence of urge incontinence among 1356 sling procedures in women over 65 [33]. A prospective study observed that 6.3% of patients developed de novo urge symptoms seven years post-TVT [34]. In a study assessing 70 women six months after they received an Advantage MUS (a type of TVT), 6% developed de novo detrusor overactivity. Of these, however, only one woman (3% of those with the condition) required antimuscarinic treatment [73]. In a retrospective study involving 52 cases of TVT, it was observed that 13% of patients developed new-onset urgency and urge incontinence. Comparing TVT and SPARC, de novo urgency rates were 40.5% and 42.4%, respectively [64]. In a prospective, observational, three-center cohort study conducted by Nilsson and colleagues, involving an initial cohort of 90 women who underwent TVT implantation, it was reported that the incidence of de novo urge urinary symptoms postoperatively was observed in 6.3% of the patients [34]. In a subsequent analysis, it was demonstrated that the incidence rates of de novo urgency following TVT and TOT procedures were 11% and 12%, respectively, with the observed difference between the two rates being statistically nonsignificant [67]. The authors previously cited in a randomized controlled study that compared the TVT and TOT reported the incidence of de novo urgency as 0% in the TVT group and 2.4% in the TOT group [65]. Kociszewski et al. and Abouassaly et al. reported 7% and 13.6% rates of de novo urge incontinence post-TVT, respectively [63,66]. A literature review by Marcelissen et al. in 2018 indicated that the occurrence of de novo and persistent urgency and urgency urinary incontinence stood at approximately 15% and 30%, respectively [74]. Furthermore, a comprehensive systematic review and meta-analysis reported that the overall incidence of de novo OAB symptoms was 11.5% in nonrandomized studies and 6.4% in randomized studies. When examining the type of MUS, de novo OAB rates were observed as 11.2% for TVT-O, 8.7% for TOT, and 9.8% for TVT, with no significant differences found between sling types [75].

In the study by Kociszewski et al., who focused solely on patients suffering from various adverse effects of MUS procedures, OAB was found to be the most common complication—it occurred in 64% of women. The authors found that women who developed OAB had a median tape-LSM distance of 1.75 mm, which was statistically significantly closer compared to cases involving other complications [35]. In the previously cited study by Viereck et al., however, a close sling–LSM distance (<3 mm) was not associated with the occurrence of OAB [68].

In a similarly structured study, published in 2012, the development of new or the worsening of existing symptoms of OAB was observed in 51% of the cohort of women with complications post MUS. Introital US was utilized to evaluate the position, dimensions, and contraction characteristics of the sling material in all the women participating in this study. It was observed that in over half of the patients who exhibited symptoms of OAB, the sling was positioned beneath the bladder neck/base [76]. In the study previously referenced by Dietz et al., concerning symptoms of OAB, correlations were identified between a tape positioned more cranially during the Valsalva maneuver and symptoms such as urge incontinence and urinary frequency. This correlation pertained to the placement of the upper tape margin, measured in relation to the infero-posterior margin of the symphysis pubis and the bladder neck. Nonetheless, these correlations were relatively weak, prompting the researchers to deduce that the success of the TVT procedure largely does not depend on its exact placement [45]. Similarly, Ghanbari et al., who recently conducted a US-based analysis of post-MUS complications in a mixed group of patients (84.8% having undergone the TOT procedure and 15.2% the TVT), reported that the distance between the tape and the bladder neck did not influence patients who experienced worsened or de novo OAB [38]. On the other hand, in their study focusing on patients who underwent MUS implantation and subsequently suffered from persistent SUI, Bogusiewicz et al. found that in two out of every three cases presenting with de novo urgency, the tapes were positioned close to the bladder neck (the authors did not specify the exact distance of the tape placement from the bladder neck) [39].

In another study, researchers conducted 2D introital US examinations on women who had undergone TVT or TOT sling procedures and were experiencing LUTS, defined as increased frequency and urgency, nocturia, poor stream, hesitancy, terminal dribbling, and urinary retention. The study revealed that the positioning of the sling in the proximal urethra might influence the onset of postoperative LUTS. Moreover, a correlation with LUTS was notably observed when the distance between the tape and urethra was 1 mm or less. However, the study did not differentiate between individual symptoms within the LUTS spectrum, making it challenging to conclusively determine if urgency was related to the tape’s position relative to the urethral length and its proximity to the urethra [77].

The previously mentioned, new meta-analysis aimed at evaluating the impact of suburethral tape placement along the urethra on surgical outcomes; data for TVT and TOT approaches were analyzed separately. No significant difference was observed in the rates of postoperative de novo urgency in either the TVT or TOT approaches, regardless of the tape’s position along the urethra [44].

Chene et al., who analyzed patients that had undergone implantation of TVT, TOT, and TVT-O, observed de novo urge incontinence in 14, 16, and 13% of patients, respectively [51]. As previously noted, the authors evaluated the angulation of the tape in various states: at rest, during the Valsalva maneuver, and at maximum retention, which involves contraction of the pelvic floor muscles. Across all three groups, a tighter angulation during retention was linked with the onset of de novo urge incontinence [51].

In summary, the positioning of the sling, especially its proximity to the bladder neck and other critical anatomical features, emerges as a significant factor influencing the occurrence of de novo OAB. While US imaging has been instrumental in evaluating sling placement and its association with OAB symptoms, findings suggest that the relationship between sling location and this adverse effect may not be straightforward. These insights underline the complexity of managing post-MUS OAB symptoms and the importance of a nuanced approach to diagnosing and addressing this complication.

### 3.4. Vaginal Exposure

Mesh sling exposure rates are estimated at approximately 2%, with variations from 0% to 8.1% reported across studies. A 2017 Cochrane Review highlighted erosion/exposure rates of 24 per 1000 for the TOT approach and 21 per 1000 for the TVT approach [9,78,79,80]. Petri et al. observed that vaginal exposures accounted for about 19% of adverse effects among those that experienced complications [76]. Based on a thorough overview, exposure rates vary significantly, with TVT slings ranging from 0% to 1.5% and TOT slings up to 10.9% [58]. A Danish study found a 2.4% exposure rate in 329 TVT cases over five years [11], while a comparison between TVT and SPARC procedures showed erosion rates of 4.8% and 10.5%, respectively [64]. In the analysis performed by Tunitsky-Bitton et al., exposure was notable in five subjects (8%), and the rate of exposure did not differ between the compared sling types (TVT, TOT, and TVT-O) [41].

Sling exposure into the vagina can be readily evaluated through clinical examination. However, the condition can also be effectively detected using US imaging [13].

As previously mentioned, in the study by Kociszewski et al., in the case of women suffering from persistent SUI and experiencing sling exposure, the sling-LSM distances were identified as 3.6 mm for persistent SUI and 4.6 mm for sling exposure, respectively, the difference being statistically significant [35]. Accordingly, in their retrospective analysis, Viereck et al. observed that patients with a sling-LSM distance of less than 3 mm had a statistically significant lower likelihood of experiencing sling exposure [68]. Similarly, Ghanbari et al., in their recent US-based analysis of post-MUS complications among a cohort that included 84.8% TOT and 15.2% TVT procedures, found that the mean sling-LSM distance in patients who experience tape exposure was statistically significantly higher compared to those in whom this complication was not diagnosed (8.80 ± 1.9 mm vs. 5.8 ± 2.0 mm) [38].

Still, Staack et al. revealed that the US is more effective in identifying urethral or periurethral erosion, but it does not enhance the detection of vaginal exposure when compared to physical examination results [81]. It is important to note that scar tissue can occasionally imitate actual slings in US imaging. The lack of color or Doppler flow within the sling can assist in distinguishing it from the adjacent native soft tissue. However, it remains unclear whether this feature can reliably differentiate the sling from fibrotic tissue [82].

In summary, the role of pelvic floor sonography in detecting vaginal exposure of MUS remains uncertain, and it presently does not appear to have a superiority over clinical examination. To fully clarify the application of pelvic floor US in this context, further investigations are necessary.

### 3.5. Sling Erosion

According to one study, the reported prevalence of MUS erosion into the urethra and/or bladder can reach up to 7.3% [83]. Other analyses, including large numbers of patients, reported incidence of erosion varying from 2.5 to 3.8% [84,85]. Results from a meta-analysis comparing TOT to TVT indicated that the rates of tape erosion or extrusion were comparable between the two surgical methods, with transobturator MUS having a 2.4% rate and retropubic MUS a 2.1% rate [86]. It is noteworthy that urethral or bladder erosion might manifest as a result of perforation during the implantation of the MUS, which could have been missed if cystoscopy was not performed [87].

**Figure 7:**
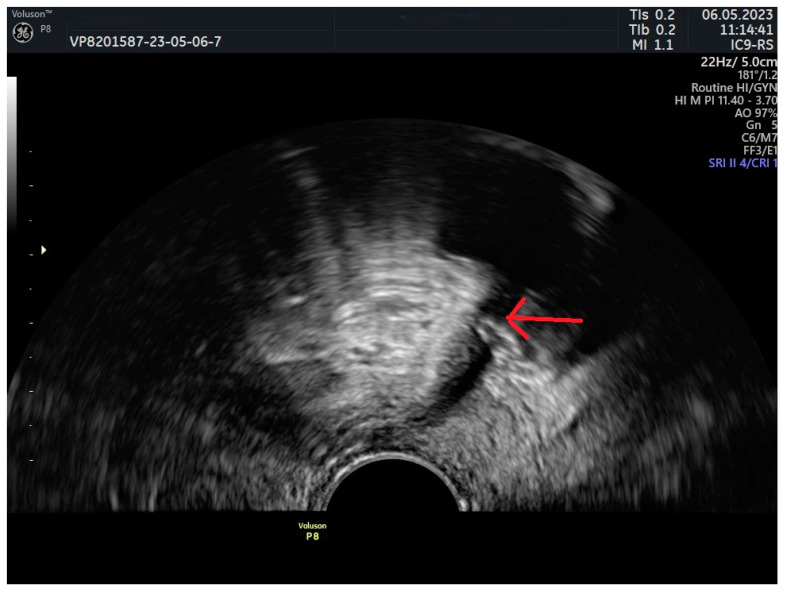
Urethral sling erosion. Red arrow—sling.

While cystourethroscopy is the standard diagnostic tool for erosion, translabial US has recently been highlighted for its potential in effectively visualizing mesh and detecting erosion [88,89] (Figure 7). In a study conducted by Viragh et al., this sonographic approach was explored for its utility in diagnosing the discussed complications of MUS. The authors identified sling erosion in 15 (8%) of 198 women who underwent surgery due to suspected MUS complications. The control group—women without documented erosion at surgery—served as a comparative baseline. In the study, the sensitivity of US was 93%, whereas specificity was 88% in detecting and localizing sling material within the lower urinary tract. The analysis revealed that US could detect erosions not visible in cystourethroscopy in a subset of patients, suggesting its significant complementary value [88].

Compared to TVT, TOT implantation is generally regarded as a procedure with a lower risk of bladder perforation, a fact that, according to some authors, eliminates the routine necessity for cystoscopy during the operation. In a recently published case report, US was demonstrated to be an effective tool in detecting an overlooked bladder perforation, which presented as bladder erosion six months following the TOT implantation. Examination by 2D US revealed the sling to be positioned above the midsection of the urethra. It was notably folded at the left posterior region of the urethra and extended partially beneath the bladder mucosa, where bladder stones had developed. Examination by 3D US further elucidated the sling’s asymmetrical placement, and ultrasonic tomography detailed how a segment of the sling traversed the bladder mucosa [87]. In the context of this publication, it should be added that stones can form within the synthetic material located in the lumen of the urethra or bladder [90]. They will manifest as hyperechoic lesions upon US examination, thus indirectly suggesting the presence of the discussed complication. This observation highlights the potential utility of US as a relevant modality in the detection of bladder or urethral tape erosions.

In summary, recent findings suggest the efficacy of pelvic floor US in detecting sling erosion, presenting a significant complementary alternative to the standard cystourethroscopy.

### 3.6. Pain

In the previously referenced analysis by Petri et al., pain at the operation site was identified as a significant adverse effect. Specifically, groin or thigh pain was associated with the TOT route, while vaginal or pelvic pain was linked to other surgical approaches, accounting for 14% of the complications observed. Additionally, it was noted that approximately 10% of women who underwent TVT experienced long-term pain. In contrast, a higher incidence of pain was observed in the TOT surgery group, where ca 34% reported this complication. A meta-analysis in which an evaluation of 11 randomized controlled trials was performed, it was also found that a significantly higher incidence of sling-related pain was experienced by patients in the TOT group (12%) compared to those in the TVT group (1.3%) (OR 9.34) [91].

Dyspareunia was observed in 6% of women who suffered complications [76]. In the study led by Anger et al., which focused on examining complications following sling surgery in female beneficiaries aged ≥65, it was found that 9.4% of women received a new diagnosis of pelvic pain [33]. Stanford et al. reported that pain, whether chronic or acute, was reported by 7.3% of patients post-MUS. Dyspareunia was found in 4.3% of patients [12]. In the retrospective analysis focusing on 100 women who experienced postoperative complications following sling procedures, it was found that a total of 40 patients endured pain related to the tape. This included dyspareunia in 29% of cases, spontaneous pain in 27%, pain while walking in 3%, and dysuria in 2% of the patients [35].

US may be essential in evaluating the condition and extent of mesh implants as well as the health of the adjacent pelvic structures. This modality is especially useful for determining whether the synthetic material is the source of pain experienced during an examination. Such diagnostic insights are invaluable for surgeons in formulating the most effective surgical strategy and ensuring patients are well-informed before consenting to procedures, which often include partial or complete removal of the mesh. However, it is important to note that imaging the retropubic or transobturator sections of the mesh using US can be challenging. This difficulty arises due to shadow artifacts created by nearby bone structures, which can obscure clear visualization. In cases where infected mesh material is suspected to be the cause of the patient’s pain, careful consideration and alternative imaging strategies may be required to accurately assess and address the issue [13].

In the study cited above, it was revealed that there was no significant difference in the tape-LSM distances nor in the position of the sling relative to urethral length when comparing women who experienced postoperative pain with those who did not [35].

**Figure 8:**
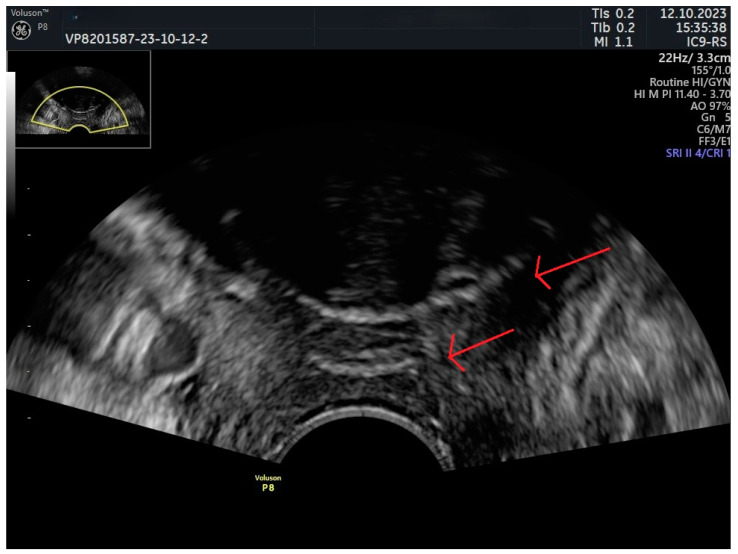
Two slings implanted in a single patient—coronal view. Red arrows—slings.

**Figure 9:**
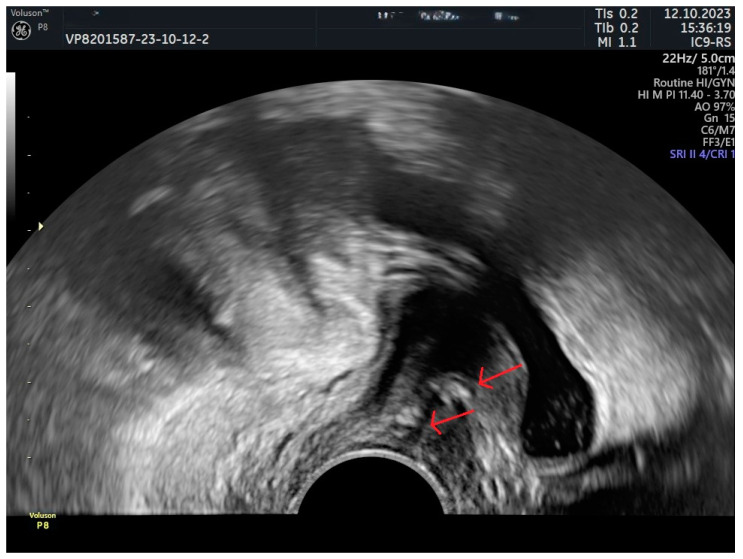
Two slings implanted in a single patient—sagittal view. Red arrows—slings.

Other authors demonstrated the utility of US in evaluating women with multiple slings, facilitating the correlation between the specific implants and pain, including dyspareunia. Through dynamic US assessment coupled with clinical examination, clinicians can identify the problematic mesh, guiding targeted interventions like excision or division of the specific implant. This approach aims to prevent symptom recurrence by ensuring that only the problematic sling is addressed. The same group suggests that US can reveal hypoechogenic areas around the MUS which, upon surgical examination, frequently indicate inflammation, infection, or fluid accumulation—potential sources of post-MUS pain [89] (Figure 8 and Figure 9).

On the other hand, in another study focusing on the association between US findings and symptoms in women experiencing complications from MUS, no US characteristics or measurements were found to correlate with the occurrence of pain or dyspareunia. This investigation involved 311 women who had been referred to a specialized clinic due to complications from their MUS procedures (both TVT and TOT). Among them, 80% reported experiencing some form of pain, either as their primary issue or alongside other symptoms. Each participant underwent both 2D perineal and 3D endovaginal US evaluations. The analyses included the sling’s distance from the urethral lumen, its position along the urethra, and its shape. No specific US characteristics or measurements correlated with the presence of pain, including dyspareunia [71]. Recently, another group yielded comparable results. In a retrospective pilot cross-sectional study, attention was directed towards the TOT, which has been associated by some studies with a higher incidence of postoperative pain compared to the TVT. The study analyzed data from patients presenting with symptomatic TOT-related pain (including inner thigh, groin, dyspareunia, or vaginal pain) or other TOT-related complications. The association between the sling’s pattern or position, as visualized by US, and related pain was investigated. It was hypothesized that variations in the sling’s pathway during insertion could result in different sling positions and angles relative to the urethra and varying tensions, potentially influencing the occurrence of neurovascular injuries and chronic pain [32,92]. Through the use of 3D endovaginal US, sling patterns were categorized into seagull (normal), lopsided, flat, and convoluted configurations. No association was found between sling-related pain and any specific sling pattern or the distance from the urethra to the sling [93].

In brief, US may emerge as an important auxiliary tool aiding in identifying the source of pain and informing surgical strategies. However, challenges in imaging certain sections of synthetic material due to shadow artifacts, and mixed findings regarding the correlation between US characteristics and postoperative pain, indicate that we cannot yet unequivocally rely on US for the diagnosis of this complication.

### 3.7. Hematoma

In a retrospective analysis, 1.9% of women undergoing the TVT procedure were diagnosed with post-surgical hematomas [63], a frequency mirrored in another study [84]. Stanford et al. found that 2% of MUS patients developed hematomas [12], while Juhl et al. observed a 1% hematoma rate post-TVT EXACT implantation [11]. Similarly, Petri et al. reported a 2% incidence of retropubic hematomas post-MUS [76]. A comparison of the TOT and TVT approaches showed hematoma rates of 0.3% in the retropubic group and 0.2% in the TOT group [94]. Another study comparing late complications between TVT and TOT noted hematoma rates requiring surgical intervention at 2.3% and 1.4%, respectively [76].

In a review focused on discussing relevant imaging techniques and demonstrating the value of imaging in postoperative urogynecological patients, the authors assert that imaging is instrumental in identifying acute or subacute perioperative complications, including hematomas, other fluid collections, infections, or vascular injuries. They note, however, that while complications like hematomas or infections in the retropubic or suprapubic space are not visible via US, magnetic resonance (MR) imaging can depict these issues [82]. Still, other researchers were able to demonstrate the usefulness of US in visualizing hematomas post-urogynecological surgeries [18].

In summary, data regarding the utilization of US for diagnosing post-MUS hematomas are limited. Based on our experience, imaging of hematomas with US is feasible, using both the vaginal and the transabdominal probe to visualize the retropubic space.

## 4. Discussion

In the studies reviewed, all authors highlighted the efficacy of sonography in identifying both urinary incontinence and complications following suburethral sling placement. For recurrent or persistent SUI, de novo OAB, and postoperative urinary retention, there is consensus on the sonographic indicators of sling positioning associated with each complication. The primary divergence lies in the examination methodology, including probe type, imaging projection, and measurement techniques for sling placement. This variation contributes to inconsistencies in findings and a lack of consensus among researchers. While the standardization of methodology is deemed less critical for identifying hematomas and erosion of synthetic material into the bladder or urethra, the universal utility of ultrasonography in diagnosing postoperative complications is undisputed. Nonetheless, there is a clear need for consensus on the technical parameters and imaging settings in ultrasonographic examinations.

Table 1 outlines prevalent complications associated with MUS procedures and the corresponding US imaging features.

## 5. Conclusions

While the existing research exhibits a degree of variability in findings, a coherent narrative unfolds. US, with its capability to offer real-time, detailed images of the pelvic floor and surrounding structures, as well as implanted synthetic materials, is an easily available imaging modality that provides clinicians with a profound understanding of the multifaceted dynamics that govern postoperative outcomes. Undoubtedly, for all the MUS complications discussed above, there is an application for this imaging method, and it seems to be indispensable in contemporary urogynecology.

## 6. Future Directions

Considering the variability in outcomes among researchers concerning the application of US in the detection of post-MUS complications, it would be beneficial to conduct additional studies including large populations of women after sling placement. These investigations would provide valuable insights into ascertaining the efficacy and precise utility of US in diagnosing complications associated with MUS procedures. Expanding this research field should focus on establishing standardized US examination protocols, enhancing the repeatability of findings, and adopting US as a routine pre- and postoperative practice. Future research should also emphasize immediate identification of complications post-surgery (presumably with the aid of US) to prevent late-stage complications, ensuring that early intervention can be implemented. Additionally, there is a need for systematic reporting of complications in relation to US findings to refine diagnostic criteria and treatment algorithms. With the anticipated advancements in sonographic technology and methodologies in the urogynecological field, we expect a significant evolution in the ability to accurately diagnose, manage, and potentially prevent complications arising from sling procedures. This progress promises to solidify US’s role as an indispensable tool in the comprehensive care of women undergoing MUS for SUI.

## 7. Limitations

In discussing the limitations of this review, it is crucial to acknowledge the heterogeneity across the research methodologies and variables. The studies utilized a variety of surgical techniques and types of MUS, including both TVT and TOT, which often originated from different manufacturers. These are two distinct surgical approaches that are characterized by different associated risks. Additionally, the criteria for assessing surgical effectiveness and complications varied among studies, incorporating both objective and subjective measures. The definitions and assessments of key outcomes such as surgical success, urinary retention, and urgency symptoms also differed. Some of the cited studies included patients in whom concurrent POP surgery had been performed, whereas some analyses of MUS complications excluded patients with coexisting OAB. The duration of follow-up also varied across the investigations. Finally, the level of surgical expertise varied among the performing surgeons.

## Figures and Tables

**Table 1:** Complications associated with MUS procedures and the corresponding US imaging features.

Sling Complication	Author, Year of Publication, Reference Number	Main Findings
Persistent/reccurent SUI	Kociszewski et al., 2017 [35]	Mean sling-LSM distance = 3.6 mm in the affected patients. Sling closer to the bladder neck in the affected patients.
	Jiang et al., 2013 [37]	Sling closer to the bladder neck in the affected patients.
	Bogusiewicz et al., 2013 [39]	Median tape position at 35% along urethral length in the affected patients.
	Flock et al., 2011 [40]	Persistent SUI associated with sling placement outside the 40–81% range of urethral length.
	Tunitsky-Bitton et al., 2015 [41]	Link between the sling position closer to the bladder neck and severity of SUI.
Urinary Retention and obstructive voiding	Kociszewski et al., 2017 [35]	Median sling-LSM distance = 1.1 mm in the affected patients.
	Reich et al., 2009 [67]	Tape-urethra distance < 2 mm in the affected patients.
	Viereck et al., 2013 [68]	Median sling-SLM distance = 1.5 mm.
	Taithongchai et al., 2021 [71]	Higher risk in patients with Group III sling shape.
	Chene et al., 2008 [51]	Tighter sling angulation in the affected patients.
De novo urgency/OAB	Kociszewski et al., 2017 [35]	Median sling-LSM distance = 1.75 mm in the affected patients.
	Petri et al., 2012 [76]	Sling close to the bladder neck in >50% of the affected patients.
	Bogusiewicz et al., 2013 [39]	Sling close to the bladder neck in 2/3 of the affected patients.
	Chene et al., 2008 [51]	Tighter sling angulation during retention in the affected patients.
Vaginal exposure	Ghanbari et al., 2022 [38]	Mean sling-LSM distance = 8.8 mm in the affected patients.
	Kociszewski et al., 2017 [35]	Mean sling-SLM distance = 4.6 mm in the affected patients.
	Viereck et al., 2013 [68]	Low risk of exposure if sling-LSM distance < 3 mm.
Sling erosion	Lou et al., 2023 [87]	A case of sling erosion into the bladder with formation of secondary bladder stones.
	Viragh et al., 2018 [88]	US could detect erosions not visible in cystourethroscopy.
Pain	Taithongchai et al., 2019 [89]	US helps identify which sling implants cause pain or dyspareunia in women with multiple slings.
Hematoma	Bogusiewicz et al., 2016 [18]	US can visualize hematomas after urogynecological surgeries.

LSM—longitudinal smooth muscle; OAB—overactive bladder; US—ultrasound; SUI—stress urinary incontinence.
